# Novel microduplication of *CHL1* gene in a patient with autism spectrum disorder: a case report and a brief literature review

**DOI:** 10.1186/s13039-016-0261-9

**Published:** 2016-06-27

**Authors:** Chunyang Li, Chunxue Liu, Bingrui Zhou, Chunchun Hu, Xiu Xu

**Affiliations:** Department of Child Healthcare, Children’s Hospital of Fudan University, Shanghai, China

**Keywords:** *CHL1* gene, 3p26.3 microduplication, Autism spectrum disorder, Developmental delay

## Abstract

**Background:**

The cell adhesion molecule L1-like (*CHL1* or *CALL*) gene is located on chromosome 3p26.3, and it is highly expressed in the central and peripheral nervous systems. The protein encoded by this gene is a member of the L1 family of neural cell adhesion molecules, and it plays a role in nervous system development and synaptic plasticity. Moreover, studies of mice have revealed that *CHL1* is a prime candidate gene for a dosage-sensitive autosomal form of mental retardation. To date, four patients with a microdeletion and two with a microduplication of 3p26.3 encompassing only the *CHL1* gene have been reported in literature.

**Case presentation:**

In the present study, we have described a 16-month-old boy with autism spectrum disorder (ASD), developmental delay and minor dysmorphic facial features. This is the first report of a duplication of 3p26.3 including only the *CHL1* gene in an ASD patient, and this duplication is the smallest reported to date in this gene. We also reviewed *CHL1* gene mutation cases and examined whether this gene has an important role in cognitive function.

**Conclusions:**

We conclude that both *CHL1* deletions and duplications are likely responsible for the patient’s impaired cognitive function, and *CHL1* may be an intriguing ASD candidate gene.

## Background

Autism spectrum disorder (ASD) is a group of neurodevelopmental disorders characterized by impairments in social interaction and verbal and non-verbal communication, as well as restricted and repetitive behaviors. In the United States, the most recent estimate as of 2010 is that 1 in every 68 children have some form of ASD [1]. Genetic factors play a prominent role in ASD pathogenesis. According to recent studies, the majority of copy number variations (CNVs) affect only one copy of a gene (which can be either deleted or duplicated), suggesting that abnormal gene dosage or expression might play a key role in susceptibility to ASD [2, 3].

The cell adhesion molecule L1-like (*CHL1* or *CALL*) gene is located on chromosome 3p26.3, and it is highly expressed in the central and peripheral nervous systems. The protein encoded by this gene is a member of the L1 family of neural cell adhesion molecules, and it plays a role in nervous system development and synaptic plasticity [4, 5]. To date, four patients with a microdeletion of 3p26.3 including only the *CHL1* gene have been reported in the literature. All of these patients presented with cognitive impairment characterized by learning and language difficulties [6–8]. Frints et al. have studied *CHL1* expression in the hippocampi of *CHL1*^+/−^ mice and have found that it is half of that observed in wild-type littermates, indicating the existence of a gene dosage effect. This group has suggested that a 50 % reduction in *CHL1* expression in the developing brain results in cognitive deficits [4]. However, less is known about the reciprocal microduplication of this gene.

To the best of our knowledge, only two families with a microduplication of 3p26.3 including only the *CHL1* gene have been described to date, including one intellectually disabled girl with epilepsy and one male patient with developmental delay (DD), symptoms of hyperactivity, a short attention span and speech delay. The duplications detected by array comparative genomic hybridization (CGH) were 1.07 and 0.85 Mb in size, respectively [4]. Here, we describe a male patient with ASD and DD for whom array CGH analysis revealed the presence of a 0.69 Mb duplication of 3p26.3. The duplication was transmitted from his normal mother and included only the *CHL1* gene.

Tassano et al. have reviewed *CHL1* gene deletion cases. In contrast, here, we review the phenotypic and molecular features of *CHL1* gene duplication cases and assess whether this gene plays an important role in cognitive function.

## Case presentation

**Fig. 1:**
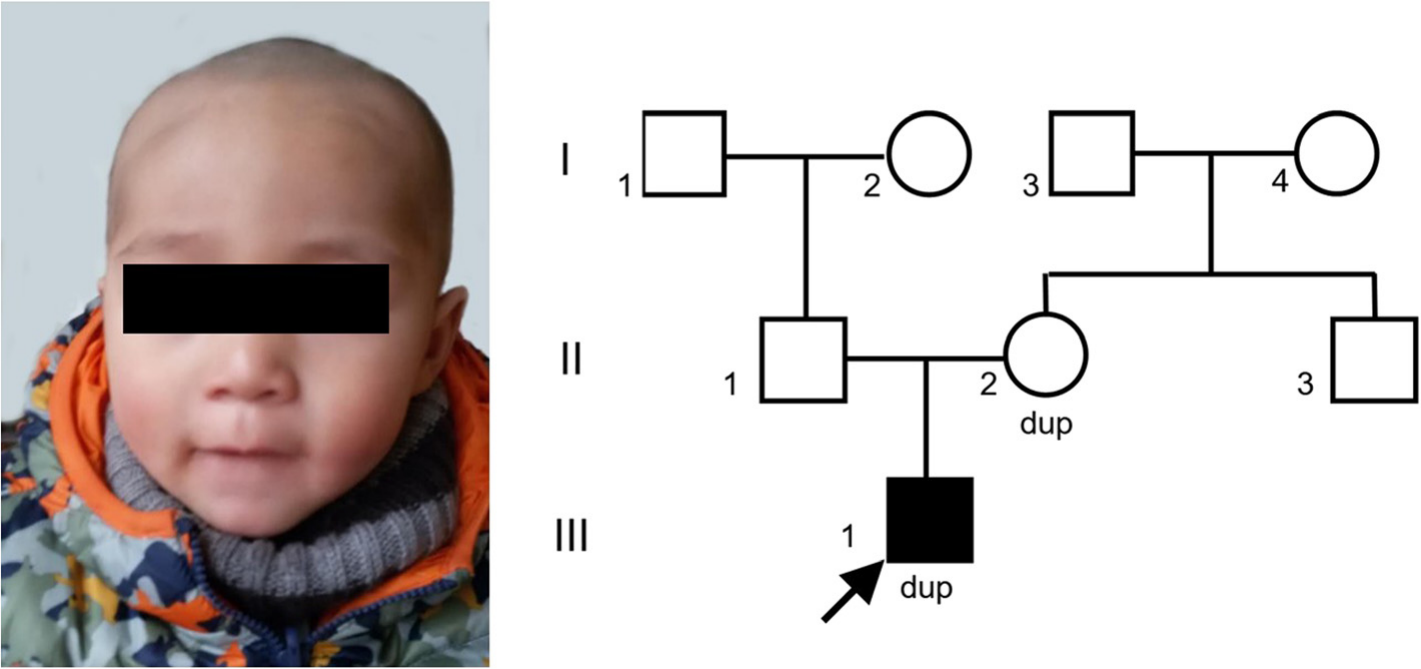
Face of the patient showing mild facial dysmorphic features and his family pedigree. Standard pedigree symbols are used; dup, duplication

The 16-month-old patient is the first child of healthy, non-consanguineous parents. He has a normal male karyotype and no family history of developmental or neuropsychiatric disorders, except for schizophrenia in his maternal grandmother. This child was born at term by caesarean section with hypoxia, and his Apgar scores were 6 and 9 at 1 and 5 min, respectively. At birth, his weight was 3450 g (50–80th percentile), and his length and head circumference were not reported. He has suffered from a feeding disorder from birth and still continues to exhibit eating refusal and vomiting problems. He had general DD and started walking at 15 months of age. His speech was delayed, with first babbling at approximately 8 months old, and he still cannot consciously generate words. He appeared to exhibit gaze avoidance and did not respond when his name was called. He preferred to look at traffic lights and exit signs. He continuously knocked the desk in the examination room with his hands or other objects. At 16 months of age, physical examination revealed the following findings: a weight of 9.95 kg (10–20th percentile), a height of 80.1 cm (20–50th percentile), and minor dysmorphic facial features, including mild hypertelorism, a short mandible and a protuberant forehead (Fig. 1). MRI scanning of his brain was unremarkable, and the EEG and fragile X screening results were also normal.

Cognitive/developmental evaluation using the Bayley Scales of Infant Development-2 showed that his cognitive and language skills, as well as his motor skills, were well behind for his age (MDI < 50; and PDI = 66). The Autism Diagnostic Observation Schedule (ADOS)-Toddler Module was used for autism screening. The results of this test revealed that the patient had a social affect score of 20, a restricted and repetitive behavior score of 5 and an overall total score of 25, indicating a likely diagnosis of moderate-to-severe ASD. His was finally diagnosed with ASD according to the Diagnostic and Statistical Manual of Mental Disorders, Fifth Edition (DSM-V) criteria.

His mother was a 37-year-old female who had considered herself healthy throughout her life. Her childhood had been unremarkable, and she did not show any DD, not even when she was young. She scored in the normal range on the Broad Autism Phenotype Questionnaire (BAPQ) [9] and Symptom Checklist-90 (SCL-90) [10].

**Fig. 2:**
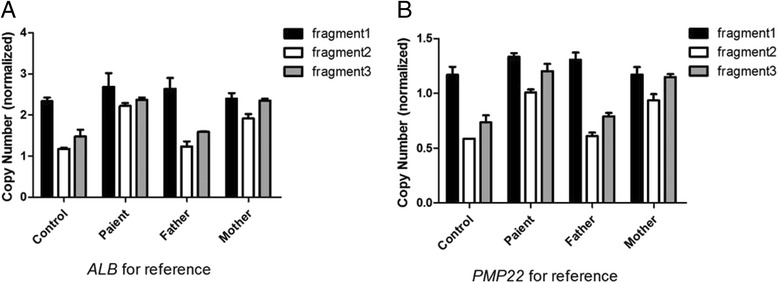
Confirmation of *CHL1* gene duplication by qPCR. **a** Results for the *ALB* reference gene. **b** Results for the *PMP22* reference gene. Fragments 1, 2, and 3 correspond to exons 4, 12 and 26 of the *CHL1* gene, respectively. The patient’s father had a normal copy number of the *CHL1* gene. The patient and his mother had a normal copy number of exon 4 but duplication of exons 12 and 26

The final patient karyotype was 46, XY, inv(9)(p12q13). Array CGH revealed a 687 kb microduplication of chromosome 3p26.3 (380,685–1,067,787) that only involved the *CHL1* gene. This duplication started at exon 5 and continued to the end of the gene. qPCR was performed, and the results verified that the gene was duplicated in the patient and that it was inherited from his normal mother. Because the duplication included the region from exon 5 to the end of the gene, exon 4 had a normal copy number. Exons 12 and 26 were duplicated (Fig. 2).

## Discussion

In the present study, we describe a 16-month-old boy with ASD, DD and minor dysmorphic facial features. This is the first report of a duplication of 3p26.3 including only the *CHL1* gene in an ASD patient, and the duplication described here is the smallest reported to date involving this gene.

The *CHL1* gene encodes a neural cell adhesion protein that belongs to the immunoglobulin superfamily. It is located on chromosome 3p26.3 and is highly expressed in the central and peripheral nervous systems. It plays a role in regulating neuronal migration and neurite overgrowth in the developing brain. Furthermore, CHL1 was expressed in the dorsal thalamus and on fibers along the thalamocortical projection in the ventral telencephalon and cortex. It could also play a vital role in thalamocortical connectivity [11]. In a study of *CHL1*-deficient (*CHL1*^−/−^) mice, researchers found that GABAergic synaptic connectivity due to the gene mutation resulted in enhanced synaptic elimination, leading to microgliosis, increased expression of interleukin-6 and the loss of parvalbumin-expressing interneurons and ultimately impaired synaptic plasticity [12]. Moreover, studies of mice have found that *CHL1* is a dosage-sensitive gene [4, 5, 12]. In humans, mutation of the *CHL1* gene has been associated with distal 3p deletion syndrome (OMIM 613792). This syndrome is a rare contiguous genetic disorder involving deletion of chromosome 3p25-p26 and is characterized by DDs, low birth weight, growth retardation, micro- and brachycephaly, ptosis, a long philtrum, micrognathia, and low-set ears [13, 14]. The *CHL1* gene is the most telomeric gene known on 3p, and it has been suggested to be candidate gene for cognitive impairment in 3p syndrome patients [4, 15].

To date, four familial cases presenting with a heterozygous deletion of chromosome 3p26.3 including only the *CHL1* gene have been reported. First, a 6-year-old boy was reported who presented with microcephaly, short stature, mild mental retardation, learning and language delays, and strabismus. His gene deletion was transmitted from his normal mother [6]. Next, two affected siblings whose deletions were transmitted from their normal father were reported who presented with microcephaly, mild mental retardation, and learning and language difficulties [7]. Finally a 12-year-old boy was reported who presented with slow physical development, microcephaly, temper tantrums, and severe learning disabilities [8]. All subjects exhibited verbal DD during the first 2 years of life and required specific therapy. The chromosomal deletions of 3p26.3 present in all four of these patients ranged from 500 Kb to 1.0 Mb in size and included only the *CHL1* gene. The deletions were inherited from an apparently unaffected parent in three cases (data not available in one case) [6].

**Fig. 3:**
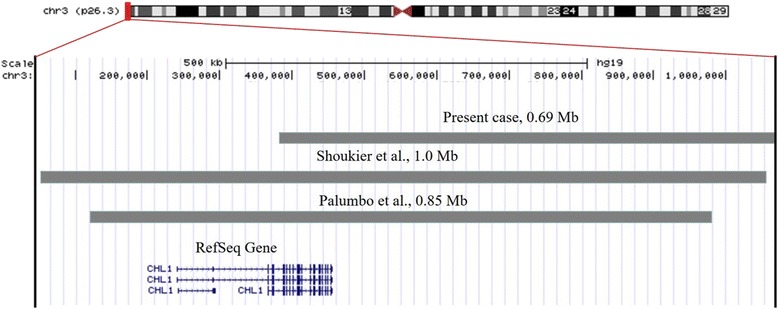
Schematic of the exact sizes and positions of chromosome 3p26.3 showing duplications reported here and other cases reported in the literature. Schematic of the 3p26.3 region displayed using the UCSC Genome browser [GRCh37/hg19 assembly; http://genome.ucsc.edu]

In addition, two families have been reported with microduplication of 3p26.3 including only the *CHL1* gene, including one intellectually disabled girl with epilepsy and one male patient with DD, symptoms of hyperactivity, a short attention span and speech delay. The phenotypic features and molecular cytogenetic findings for these two patients, as well as our patient, are shown in Table 1, and the exact sizes and positions of duplications of these three cases are shown in Fig. 3. All of the patients showed DD/intellectual disability and language delay. Moreover, 6 other similar cases have been reported in the Decipher Database [https://decipher.sanger.ac.uk]. Among these 6 cases, one patient exhibited a 731.32 kb duplication on 3p26.3, containing the entire *CHL1* gene, that was characterized by global DDs, a retinal abnormality, feeding difficulties, hypophosphatemia, and scrotal hypoplasia (n.279556). One patient with a duplication of the entire *CHL1* gene together with another variant on chromosome 7 was characterized by intellectual disability (n.287862). Another four patients displayed duplication of the first exon on *CHL1* gene and presented intellectual disability or global DD with or without other phenotypic anomalies (n.288373, n.289009, n.291512, and n.289754). Because the information available in the Decipher Database is not comprehensive, we used the cases that are reported in the literature for discussion.

**Table 1:** Comparison of clinical and molecular findings associated with 3p26.3 duplication, including only the *CHL1* gene, between the present study and previous studies

Subject	Present case	Shoukier et al. [26]	Palumbo et al. [27]
Sex	F	M	F
Age	1 years and 4 months	16 years	2 years and 3 months
Duplication size	0.69 Mb	1.0 Mb	0.85 Mb
Coordinates (hg19)	380,685–1,067,787	48,914–1,054,209	125,931–975,649
Inheritance	Maternal	Maternal	*De novo*
Pregnancy condition	Hypoxia	Normal	Normal
Delivery	Term	Term	Term
Family history	Maternal grandmother with schizophrenia	No family history	No family history
Weight (g)	9.95 kg (10–20th percentile)	57 kg (50th percentile)	15 kg (75–90th percentile)
Height (cm)	80.1 cm (20–50th percentile)	157 cm (25th percentile)	96 cm (90–97th percentile)
Dysmorphic facial features	Mild hypertelorism, short mandible and protuberant forehead	No dysmorphic facial features	Minor dysmorphic facial features, including mild hypertelorism, down-slanting, long palpebral fissures with eversion of lateral third of lower eyelids, long philtrum, thin upper lip, and mildly prominent ear lobes
Age at walking	15 months	15 months	12 months
Verbal DD	+	+	+
Seizures	-	+	-
DD/ID	+	+	+
Feeding disorder	+	-	-
ASD-related features	+	-	-
Hyperactivity/attention deficit	-	-	+
Brain MRI	Normal	Normal	Normal
EEG	Normal	Multifocal sharp waves and sharp and slow-wave complexes	Normal
Fragile X screening	Normal	Normal	Not reported

*M* male, *F* female, *+* present, *−* absent, *DD* developmental delay, *ID* intellectual disability

Most of the genetic aberrations in these seven patients were transmitted from a normal parent (three were maternal, two were paternal, one was *de novo* and no data were available in one case). These observations are suggestive of incomplete penetrance. In addition, Chl1^+/−^ mice have been demonstrated to have a phenotypic spectrum ranging from wild-type to knockout behaviors [4]. Another possible explanation would be the two-hit model [16]. Other undetected CNVs might exist within the Array CGH in our patient; these mutations together with the *CHL1* duplication could have caused the abnormalities in the child but not affected his mother. In addition, apart from the microarray-analyses, microscopically visible CNVs (CG-CNVs) might be another possibility [17]. Moreover, an apparently unaffected parent who carries the deletion could also have subtle associated phenotypic features that become evident upon further clinical evaluation. Other possibilities that may account for the phenotypic variability among these patients include differences in genetic background, epigenetic phenomena, expression or regulatory variation, and the unmasking of recessive variants located on the other allele [7, 18].

Because the *CHL1* gene plays an important role in regulating neuronal migration and neurite overgrowth in the developing brain and all seven patients with a *CHL1* gene mutation showed DD, we conclude that both *CHL1* deletion and duplication are likely to result in impaired cognitive function in patients. One interpretation of the gene balance hypothesis is that under- and overexpression phenotypes are identical, or at least similar, because both deletions and duplications disrupt an identical multimeric regulatory protein complex (by disruption of the stoichiometry of protein subunits encoded by the associated genes) [19]. The relationship between gene dosage and phenotype is complex. Conrad and Antonarakis have proposed three alternatives to explain this relationship that are each characteristic of certain categories of genes. Based on the reports in the literature, we hypothesize that the *CHL1* gene corresponds with the third alternative, which involves certain functional gene classes enriched in transcriptional regulators and signaling molecules that cause phenotypic changes when both under- and overexpressed (haploinsufficiency and pathogenic gene duplication). Both deletions and duplications are known to occur within the same chromosomal region in other diseases, for example, the progressive neurodegenerative disorder Rett syndrome, which almost exclusively affects females in its classical form and is due to *MECP2* haploinsufficiency. Severe mental retardation and neurological symptoms with features of Rett syndrome in males can also be caused by *MECP2* duplication [19].

With regard to association of the *CHL1* gene with ASD, Weehi et al. have identified a patient with a 3p26.3 microduplication encompassing part of the *CHL1* gene as well as the *CNTN6* gene who presented with motor and speech DDs and some autistic features. Features in this patient suggestive of ASD included repetitive activities, preoccupation with spinning wheels, resistance to changes in routine, and heightened sensitivity to people touching his legs. Overall, however, he was not considered to have ASD [20]. Moghadasi et al. studied the clinical consequences of a terminal deletion of the short arm of chromosome 3 in four generations of a family. They detected a 2.9 Mb deletion in the short arm of chromosome 3 in 7/13 family members that contained the candidate genes *CHL1* (OMIM 607416), *CNTN4* (OMIM 607280), and *CNTN6* (OMIM 607220). The sister of the index patient had autism, speech delay, and learning difficulties [21]. In addition, 3p26.3 microduplication has been reported in a study of CNVs in extended ASD families, but this aberration did not affect any genes and was located 109 Kb upstream of *CHL1* [22]. In the present study, we report a terminal duplication of 3p26.3 confined to the *CHL1* gene in a boy with ASD. ASD is a group of neurodevelopmental disorders. The etiology and pathogenesis are still unknown. However, some studies have shown that the pathogenesis of ASD during early development of the brain could be relevant to disruptions of serotonin signaling [23]. CHL1 is a modulator of the serotonergic system; the signal transduction pathways are regulated by constitutive activation of the serotonin 2c (5-HT2c) receptor by CHL1 [24]. Another study discussed the genome-wide expression profiling of human lymphoblastoid cell lines and found that CHL1 is a putative selective serotonin reuptake inhibitor (SSRI) response biomarker [25]. Therefore, *CHL1* may be an intriguing ASD candidate gene, and it is likely responsible for the cognitive function impairments of ASD patients with a *CHL1* gene mutation.

## Conclusion

In the present study, we have described a 16-month-old boy with ASD, DD and minor dysmorphic facial features. This is the first report of a duplication of 3p26.3 confined to the *CHL1* gene in an ASD patient, and it is the smallest reported duplication to date. We have also reviewed *CHL1* gene mutation cases from the literature and examined whether this gene plays an important role in cognitive function. Although some clinical symptoms differ among these patients, such as epilepsy and some autistic features, all of the subjects showed impaired cognitive function. Thus, both heterozygous deletions and duplications of the *CHL1* gene may be considered in the etiology of a new emerging syndrome characterized by DD and other possible symptoms. Because the number of reports is limited, the identification of new cases and further functional studies are required to confirm the exact function of the *CHL1* gene in cognitive development.

## Methods

Array CGH was performed on DNA extracted from the peripheral blood of the patient using commercially available arrays (Agilent SurePrint G3 Human CGH Microarray 1 × 1 mol/l, Agilent Santa Clara, California, USA). The UCSC Genome Browser database (GRCh37/hg19, Feb. 2009) was used as reference of structural variations identified in healthy human samples.

Real-time quantitative polymerase chain reaction (qPCR) was performed (SuperReal PreMix Plus, TIANGEN, China) to verify the presence of the *CHL1* microduplication in this patient and to determine whether it was inherited. Three pairs of primers were designed using IDT, an online primer-designing tool (https://www.idtdna.com/Primerquest), for exons 4, 12 and 26 of *CHL1* gene transcript variant 1. Relative quantification was performed using a standard curve against control amplicons of the *ALB* and *PMP22* genes according to the manufacturer’s instructions (LightCycler® 480 Instrument, Roche, Germany).

## Abbreviations

ADOS, autism diagnostic observation schedule; ASD, autism spectrum disorder; BAPQ, broad autism phenotype questionnaire; CGH, comparative genomic hybridization; *CHL1*, cell adhesion molecule L1-like; CNVs, copy number variations; DD, developmental delay; DMS-V, diagnostic and statistical manual of mental disorders, fifth edition; SCL-90, symptom checklist-90; qPCR, real-time quantitative polymerase chain reaction
